# Association between multimorbidity and mean platelet volume in diabetic patients with acute myocardial infarction

**DOI:** 10.1007/s00592-017-1079-6

**Published:** 2017-11-30

**Authors:** Bartosz Hudzik, Ilona Korzonek-Szlacheta, Janusz Szkodziński, Radosław Liszka, Andrzej Lekston, Barbara Zubelewicz-Szkodzińska, Mariusz Gąsior

**Affiliations:** 1 Third Department of Cardiology, Silesian Center for Heart Disease, School of Medicine with the Division of Dentistry in Zabrze, Medical University of Silesia, Katowice, Poland; 2 Department of Nutrition-Related Disease Prevention, School of Public Health in Bytom, Medical University of Silesia, Katowice, Poland

**Keywords:** Multimorbidity, Diabetes mellitus, Myocardial infarction, Mean platelet volume

## Abstract

**Aims:**

Diabetes mellitus (DM) is one of the most frequently detected conditions in multimorbid disease clusters. Platelet activation is one of the key mechanisms underlying atherothrombosis in acute myocardial infarction. Available data link mean platelet volume (MPV) to poor prognosis not only in cardiovascular and non-cardiovascular disease. Given the lack of research data on the association between disease clusters and MPV, we have set out to investigate the link between multimorbidity and MPV in diabetic patients with acute myocardial infarction.

**Methods:**

A total of 277 patients with DM and STEMI undergoing primary percutaneous coronary intervention were enrolled. Based on the number of comorbidities the study population was divided into two groups: group 1 (*N* *=* 58) with ≤ 1 comorbidity and group 2 (*N* *=* 219) with ≥ 2 comorbidities. A subanalysis was performed within the multimorbidity group: group 2A with two or three comorbidities (*N* *=* 156) and group 2B with at least four comorbidities (*N* *=* 63).

**Results:**

In the study population, 15.9% of patients had one comorbidity, and 22.0, 34.3, and 22.7% of patients had two, three, or at least four comorbid conditions, respectively. Both MPV and PDW were elevated in multimorbid patients (9.3 vs 10.8 fl and 9.5 vs 10.3 fl, respectively). The highest platelet volume indices were observed in patients with at least four comorbid conditions. There was a moderate positive correlation between MPV and the total number of comorbidities, the number of CVD comorbidities, and the number of non-CVD comorbidities.

**Conclusions:**

These findings indicate that multimorbidity is associated with an increase in platelet volume indices. MPV values increased with the increasing number of comorbid conditions. Importantly, MPV values were elevated in some, but not all CVD and non-CVD conditions.

## Introduction

Type 2 diabetes mellitus (DM) has become one of the more prevalent conditions worldwide in the past few decades [1]. DM is a chronic condition that is associated with a plethora of complications. This, in turn, results in high morbidity and mortality. Diabetic patients have at least one coexisting chronic disease and approximately 40% have at least three [2, 3]. DM is one of the most commonly measured diseases in studies of multimorbidity, and more importantly it is one of the most frequently detected conditions in multimorbid disease clusters [4, 5]. Patients with diabetes mellitus who are hospitalized for other health problems may have increased risk of in-hospital death and longer hospital stay. For this reason, diabetes should be promptly recognized upon admission and properly managed [6]. That said, the risk of cardiovascular and other macrovascular complications varies substantially among diabetic patients [7, 8]. Diabetes is a major contributor to the development of cardiovascular disease (CVD), stroke, chronic kidney disease, non-traumatic lower limb amputations, and blindness. Growing number of chronic diabetes-related complications and comorbid conditions have been associated with poor metabolic control, less optimal disease management, higher health service utilization, impaired physical functioning, and worse outcomes [5, 9–11].

Platelet activation is one of the key mechanisms underlying atherothrombosis in acute myocardial infarction [12]. Platelets of diabetic patients are characterized by dysregulation of several signaling pathways, which leads to increased platelet reactivity. This may play a role not only in the higher risk of developing acute myocardial infarction and the worse outcomes observed in DM, but also in the larger proportion of DM patients with inadequate response to antiplatelet agents compared with non-DM patients [13]. The methods of testing platelet activity may be very time consuming, expensive, and technically difficult [14]. The mean platelet volume (MPV) is a readily available parameter with routine blood counts by automated hemograms and, therefore, is an attractive index to study in clinical scenarios [15]. Available data show that increased MPV is linked to the presence of risk factors for cardiovascular disease including among others: diabetes mellitus [16], impaired fasting glucose [17], hyperlipidemia [18], and metabolic syndrome [19]. MPV is a marker of platelet size and activity and has been linked to poor prognosis following St elevation myocardial infarction (STEMI) [20, 21]. In this sense, regular glycemic control could result in lowering disease burden, especially when DM itself and glycemia have a great impact on platelet function [22–24].

Available data link MPV to poor prognosis not only in cardiovascular disease, but also in malignant tumors, chronic kidney disease, lung disease, kidney disease, and rheumatoid condition among others [25–28]. Given lack of research data on the association between disease clusters and MPV, we have set out to investigate the link between multimorbidity and MPV in diabetic patients with acute myocardial infarction.

## Materials and methods

The study conforms to the Declaration of Helsinki. Informed consent for data analysis was obtained from the patients according to the Polish law on patients’ rights regarding data registration. Approval for analyzing recorded data was waived by the local bioethics committee on human research given the retrospective nature of the study. Patients with DM admitted with diagnosis of ST elevation myocardial infarction (STEMI), within 12 h from symptom onset were enrolled in the study. A total of 277 patients with DM and STEMI undergoing primary percutaneous coronary intervention (PCI) were enrolled. Based on the number of comorbidities the study population was divided into two groups: **group 1 (** ***N*** ***=*** **58)** with ≤ 1 comorbidity and **group 2 (** ***N*** ***=*** **219)** with ≥ 2 comorbidities. Furthermore, a subanalysis was performed within the multimorbidity group: **group 2A** with two or three comorbidities **(** ***N*** ***=*** **156)** and **group 2B** with at least four comorbidities **(** ***N*** ***=*** **63).**

All patients received loading doses of antiplatelet medications (aspirin, clopidogrel) before admission to our hospital (either in the referring hospital or ambulance) according to the guidelines.

Venous blood samples were collected on admission in standardized dipotassium ethylenedinitrotetraacetic acid (EDTA) tubes. The samples were tested within 30 min of collection to minimize variations due to sample aging. Platelet indices were measured as part of the automated CBC using a Sysmex XS1000i and XE2100 (Sysmex Corporation, Kobe, Japan). The definitions of STEMI and DM have been described in detailed previously [29]. Coronary angiography and percutaneous coronary interventions were performed using standard protocols and guidelines. A culprit lesion was described in the presence of an acute occlusion, intraluminal filling defects (or thrombus), ulcerated plaques, dissection, or intraluminal flaps.

We reviewed the medical records of patients who were admitted with a diagnosis of STEMI and reviewed each of their hospital charts. Comorbidities in the present study were defined as those chronic conditions that were previously diagnosed, and had been documented, in the medical history section of reviewed hospital charts, or that may have been newly diagnosed during the patient’s hospital stay. Comorbidities were grouped into two categories: (a) CVD comorbidities which included hypertension (HTN), atrial fibrillation (AF), heart failure (HF), hyperlipidemia, stroke, and peripheral artery disease (PAD), and (b) non-CVD criteria which included chronic obstructive pulmonary disease (COPD), asthma, cancer, anemia, peptic ulcer/GI bleeding, chronic kidney disease (CKD) stage ≥ 3 (estimated glomerular filtration rate below 60 mL/min/1.73 m^2^), thyroid disorders (hypo-/hyperthyroidism, goiter), depression, and connective tissue disease (CTD). The afore-mentioned CVD and non-CVD were selected based on the findings of previous reports that have pointed to the association of these conditions with outcomes following AMI [30–33].

We adopted the most widely used definition of multimorbidity—that is, the coexistence of multiple chronic diseases and medical conditions in the same individual (defined as two or more conditions) [34–36]. We used the World Health Organization definition of chronic disease, which is “health problems that require ongoing management over a period of years or decades” [37].

### Statistical analysis

Quantitative data are presented as means ± standard deviations (SD) or medians with interquartile ranges (lower and upper quartiles). Qualitative data are presented as frequencies. The Shapiro–Wilk test was used to determine whether random samples came from a normal distribution. The Chi-square test with Yates’ correction was used to compare categorical variables. The unpaired *t* test was used to compare normally distributed continuous variables between groups. The Mann–Whitney *U* test was used to compare continuous variables with a distribution other than normal. One-way analysis of variance (ANOVA) and Kruskal–Wallis ANOVA tests were used to compare continuous variables between groups 1, 2A, and 2B for variables normally and not normally distributed, respectively. The relationship between MPV and clinical/laboratory variables was evaluated by Spearman’s rank correlation coefficient. A value of two tailed *P* < 0.05 was considered significant.

## Results

**Fig. 1:**
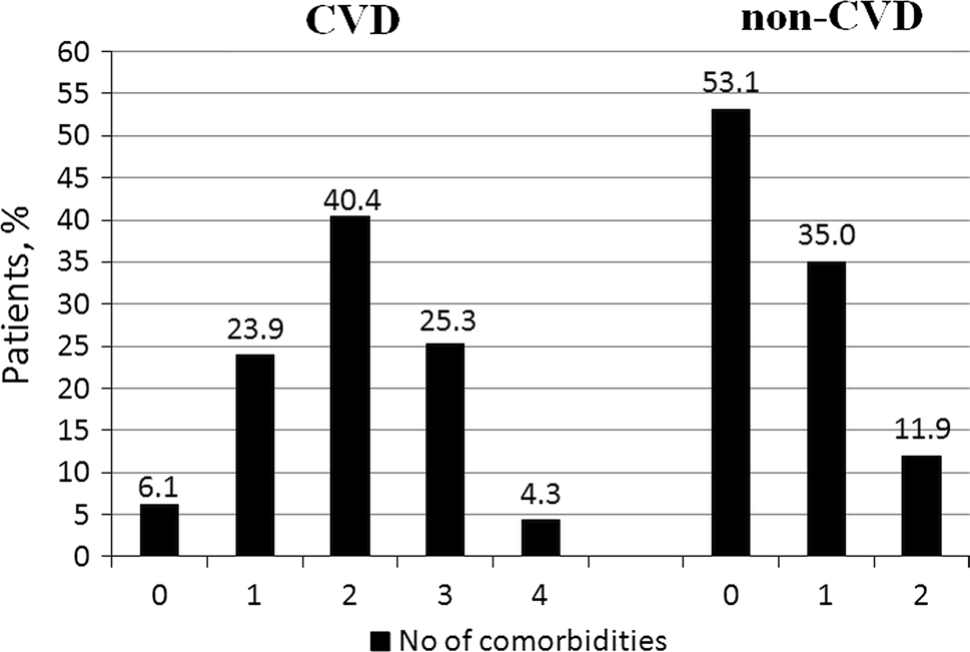
Distribution of the number of comorbid cardiovascular (CVD) and non-cardiovascular (non-CVD) conditions

**Fig. 2:**
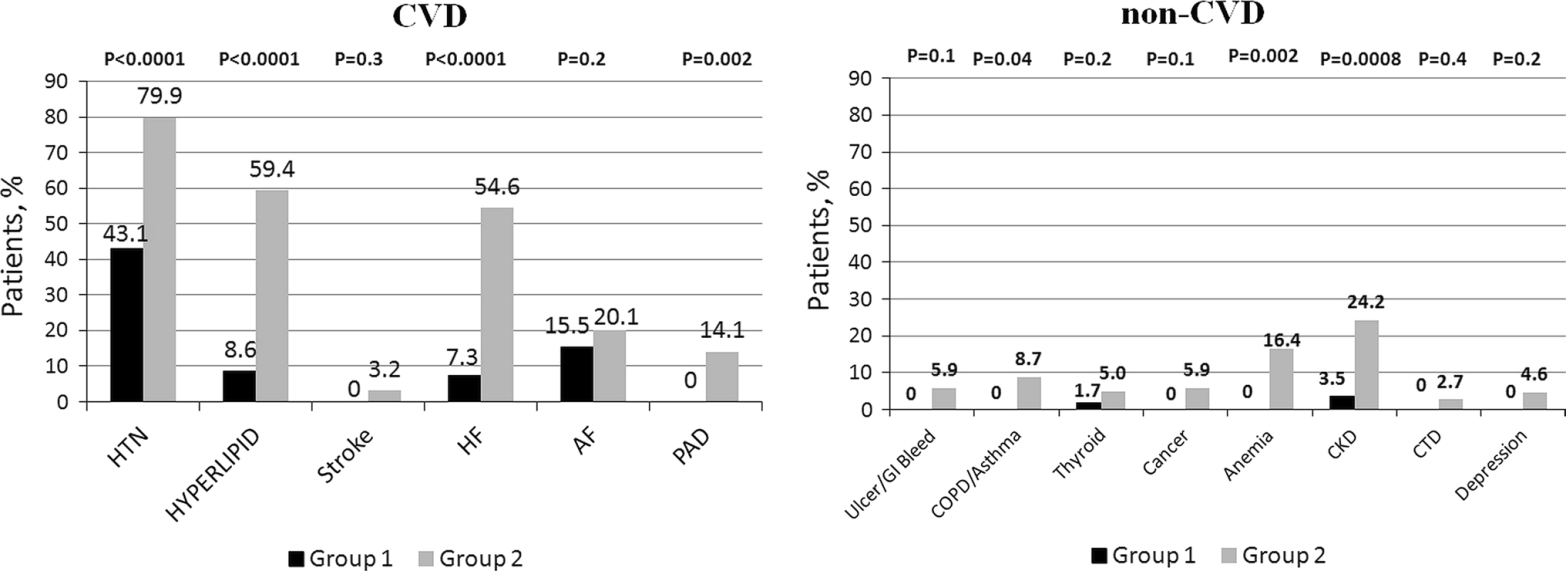
Prevalence of selected cardiovascular (CVD) and non-cardiovascular (non-CVD) comorbidities

The median number of comorbidities was three. In the study population, 15.9% of patients had one comorbidity and 22.0, 34.3, and 22.7% of patients had two, three or at least four comorbid conditions, respectively. The number of comorbid cardiovascular (CVD) and non-cardiovascular (non-CVD) conditions is depicted in Fig. 1. The two study groups differed with respect to the use of insulin and metformin. Patients with multimorbidity had more impaired left ventricular systolic function and required longer in-hospital stay (7.5 vs 9 days *P* = 0.04) (Table 1). Figure 2 depicts the prevalence of selected CVD and non-CVD comorbidities among patients with STEMI and DM. Angiographic characteristics were similar in all study groups (Table 2). Multimorbid patients had elevated levels of total cholesterol and LDL cholesterol (Table 3).

**Table 1:** Patients’ baseline and clinical characteristics

	Group 1 *N* *=* 58	Group 2 *N* *=* 219	*P*	Group 1 *N* *=* 58	Group 2A *N* *=* 156	Group 2B *N* *=* 63	*P*
Age, years (mean ± SD)	63 ± 8	64 ± 10	0.5	63 ± 8	63 ± 10	65 ± 10	0.6
Men, *N* (%)	37(63.8%)	126(57.5%)	0.4	37(63.8%)	98(62.8%)	28(44.4%)	0.03
Prior myocardial infarction, *N* (%)	11(19.0%)	64(29.3%)	0.1	11(19.0%)	42(27.1%)	22(34.9%)	0.1
Smoking, *N* (%)	12(20.7%)	26(11.9%)	0.04	12(20.7%)	20(12.8%)	6(9.5%)	0.04
Time from symptom onset, hours [median (interquartile range)]	5.0(3.0–6.0)	4.0(3.0–7.0)	0.8	5.0(3.0–6.0)	4.0(3.0–7.0)	4.0(3.0–8.0)	0.6
Cardiogenic shock, *N* (%)	8(13.8%)	34(15.5%)	0.7	8(13.8%)	25(16.0%)	9(14.3%)	0.9
Insulin^a^, *N* (%)	24(41.4%)	138(63.8%)	0.002	24(41.4%)	91(58.3%)	47(74.6%)	<0.001
Metformin^a^, *N* (%)	34(58.6%)	88(40.2%)	0.02	34(58.6%)	65(41.7%)	23(36.5%)	0.04
Sulfonylureas^a^, *N* (%)	19(32.8%)	70(32.0%)	0.8	19(32.8%)	47(30.1%)	23(36.5%)	0.5
HbA1c, (%)	7.7(6.9–8.5)	7.5(6.9–8.0)	0.3	7.7(6.9–8.5)	7.6(7.0–8.3)	7.5(6.8–8.4)	0.3
LVEF, (%) [median (interquartile range)]	47(45–51)	40(35–45)	<0.001	47(45–51)	42(35–45)	39(32–42)	<0.001
Hospital stay, days [median (interquartile range)]	7.5(6–10)	9(6–12)	0.04	7.5(6–10)	9(6–12)	10(6–13)	0.055

*SD* standard deviation; *LVEF* left ventricular ejection fraction

^a^Some patients were on more than one hypoglycemic agent

**Table 2:** Angiographic findings

	Group 1 *N* *=* 58	Group 2 *N* *=* 219	*P*	Group 1 *N* *=* 58	Group 2A *N* *=* 156	Group 2b *N* *=* 63	*P*
Multivessel CAD, N (%)	28 (48.3%)	111 (50.7%)	0.8	28 (48.3%)	77 (49.4%)	34 (54.0%)	0.2
*Initial TIMI flow, N* (*%*)							
0	35 (60.3%)	147 (67.5%)		35 (60.3%)	103 (66.0%)	44 (71.0%)	
1	12 (20.7%)	36 (16.5%)	0.6	12 (20.7%)	30 (19.2%)	7 (9.7%)	0.4
2	11 (19.0%)	36 (16.5%)		11 (19.0%)	23 (14.8%)	12 (19.3%)	
3	0 (0%)	0 (0%)		0 (0%)	0 (0%)	0 (0%)	
*Final TIMI flow, N* (*%*)							
0	2 (3.4%)	13 (5.9%)		2 (3.4%)	6 (3.8%)	4 (6.3%)	
1	1 (1.7%)	2 (0.9%)	0.4	1 (1.7%)	2 (1.3%)	0 (0%)	0.2
2	3 (5.2%)	20 (9.1%)		3 (5.2%)	16 (10.2%)	3 (4.8%)	
3	52 (89.9%)	184 (84.0%)		52 (89.9%)	132 (84.6%)	56 (88.9%)	

*CAD* coronary artery disease, *TIMI* thrombolysis in myocardial infarction

**Table 3:** Laboratory findings

	Group 1 *N* *=* 58	Group 2 *N* *=* 219	*P*	Group 1 *N* *=* 58	Group 2A *N* *=* 156	Group 2b *N* *=* 63	*P*
Leukocytes (10^3^/mm^3)^	14.5 ± 4.8	13.9 ± 5.6	0.6	14.5 ± 4.8	14.2 ± 6.0	13.2 ± 5.0	0.6
Erythrocytes (10^6^/mm^3^)	4.5 ± 0.5	4.5 ± 0.6	0.9	4.5 ± 0.5	4.5 ± 0.6	4.5 ± 0.8	0.9
Hemoglobin (g/dL)	14.5 ± 1.3	13.9 ± 1.6	0.4	14.5 ± 1.3	14.2 ± 3.0	13.4 ± 2.4	0.4
Hematocrit (%)	42 ± 5	41 ± 5	0.5	42 ± 5	41 ± 5	40 ± 5	0.5
Platelet count (10^3^/mm^3^)	228 ± 66	217 ± 70	0.7	228 ± 66	219 ± 63	212 ± 82	0.9
Admission glycemia (mmol/l)	9.7 ± 2.7	9.3 ± 3.8	0.7	9.7 ± 2.7	9.4 ± 3.9	9.1 ± 3.8	0.4
Total cholesterol (mmol/l)	4.7(4.4–5.8)	5.7(4.9–7.1)	0.01	4.7(4.4–5.8)	6.0(4.8–7.3)	5.6(5.0–6.6)	0.02
HDL cholesterol (mmol/l)	1.4(1.1–1.7)	1.3(1.1–1.6)	0.8	1.4(1.1–1.7)	1.4(1.0–1.7)	1.2(1.1–1.6)	0.6
LDL cholesterol (mmol/l)	3.0(2.5–3.9)	4.2(3.2–4.6)	0.01	3.0(2.5–3.9)	4.6(3.6–5.0)	3.9(3.2–4.6)	0.02
Triglycerides (mmol/l)	1.1(0.8–1.7)	1.2(0.9–1.8)	0.7	1.1(0.8–1.7)	1.1(0.8–1.7)	1.3(0.9–1.9)	0.5
Serum creatinine (μmol/l)	83(76–101)	89(77–114)	0.5	83(76–101)	86 (77–99)	84(76–108)	0.3
eGFR (ml/min per 1.73 m^2^)	75(67–87)	70(60–85)	0.4	75(67–87)	72(62–87)	69(50–80)	0.9

**Fig. 3:**
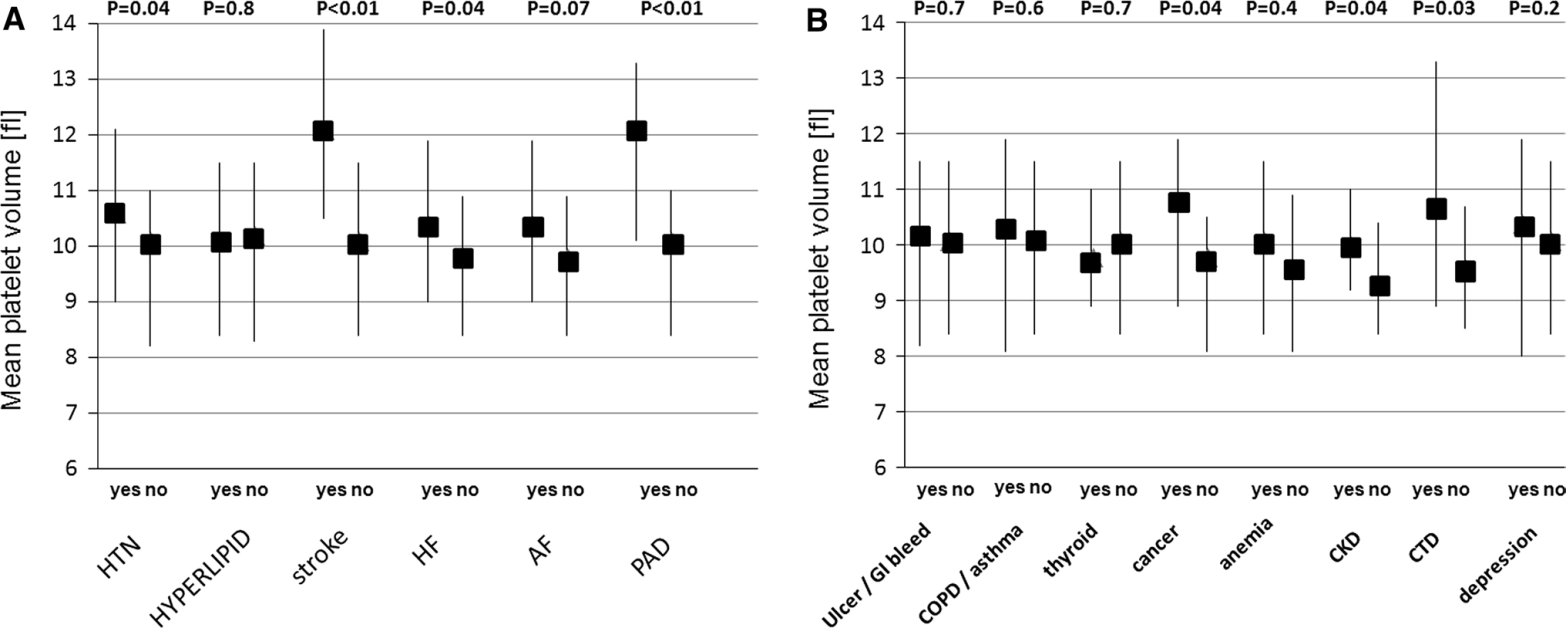
Median (interquartile range) values of mean platelet volume across the spectrum of cardiovascular disease (CVD) and non-CVD conditions

If we now turn to platelet volume indices, both MPV and PDW were elevated in multimorbid patients (9.3 vs 10.8 fl and 9.5 vs 10.3 fl, respectively). Similarly, MPV-to-platelet count (MPV/PC) ratio was increased in patients with multimorbidity (Table 4). What is more, the highest platelet volume indices were observed in patients with at least four comorbid conditions (Table 4). Figure 3 demonstrates median MPV values across all studied CVD and non-CVD comorbid conditions. Table 5 provides the correlations between the studied platelet volume indices and the number of comorbid conditions. There was a moderate positive correlation between MPV and the total number of comorbidities, the number of CVD comorbidities, and the number of non-CVD comorbidities. Unlike MPV, PDW correlated only with the number of CVD comorbidities.

**Table 4:** Platelet volume indices

	Group 1 *N* *=* 58	Group 2 *N* *=* 219	*P*	Group 1 *N* *=* 58	Group 2A *N* *=* 156	Group 2b *N* *=* 63	*P*
MPV (fl)	9.3(8.1–11.0)	10.8(8.9–12.3)	0.03	9.3(8.1–11.0)	10.5(8.7–12.0)	11.1(9.1–12.5)	0.02
PDW (fl)	9.5(8.5–10.4)	10.3(8.9–11.1)	0.03	9.5(8.5–10.4)	10.1(8.8–10.9)	10.6(9.1–11.4)	0.04
MPV/PC (fl/10^4^)	0.408(0.388–0.435)	0.497(0.412–0.513)	0.02	0.408(0.388–0.435)	0.453(0.399–0.501)	0.517(0.432–0.541)	0.03

*MPV* mean platelet volume, *MPV/PC* mean platelet volume to platelet count ratio, *PDW* platelet distribution width

**Table 5:** Correlation between platelet volume indices and the number of comorbidities

	MPV		PDW		MPV/PC	
	*R*	*P*	*R*	*P*	*R*	*P*
Total number of comorbidities	0.45	0.03	0.14	0.2	0.18	0.04
Number of cardiovascular comorbidities	0.48	0.02	0.23	0.03	0.20	0.04
Number of non-cardiovascular comorbidities	0.52	0.04	0.15	0.3	0.10	0.4

*MPV* mean platelet volume, *MPV/PC* mean platelet volume to platelet count ratio, *PDW* platelet distribution width

## Discussion

We investigated the effect of multimorbidity on MPV in diabetic patients with acute myocardial infarction. There are several key findings of our study. First and foremost, multimorbid patients had increased platelet volume indices, including MPV. Second, MPV values increased with the increasing number of comorbid conditions with the highest platelet volume indices were observed in patients with at least four comorbid conditions. And finally, MPV values were elevated in some, but not all CVD and non-CVD conditions. More importantly, it appeared that rather disease clusters than the specific disease type were associated with increased platelet volume indices.

We found that multimorbidity was highly prevalent among diabetic patients with STEMI. This finding is consistent with previous reports [2, 3]. Specific conditions have been previously linked to elevated MPV; however, we did not find any prior studies assessing the effect of multimorbidity on MPV.

Based on recent studies, inflammation—a biological mechanism—appears to be a common mechanism in numerous chronic illness, especially multimorbidity. Friedman et al. [38] studied 1229 patients from the Survey of Mid-Life in the United States (MIDUS). They assessed direct relationships between multimorbidity and activities of daily living as well as indirect associations with a latent variable for inflammation (indicated by circulating levels of interleukin 6, C-reactive protein, and fibrinogen) as a mediator. The authors reported that after adjustment for potential confounds, multimorbidity was positively associated with inflammation (*P* < 0.001) and functional limitations (*P* < 0.001), and inflammation partially mediated the link between multimorbidity and functional limitations (*P* < 0.01) [38]. Moreover, a wide range of population-based studies have shown that inflammatory state is higher in adults with single chronic medical disease [39–41] and seems to progress further with every additional chronic illness in adults with multimorbidity [42, 43]. Fabri et al. [42] studied 1018 participants of the InCHIANTI Study and reported that higher baseline IL-6 concentrations and steeper increase of IL-6 concentrations were significantly and independently associated with a abrupt increase in multimorbidity over time (*P* < 0.001 and *P* = 0.003, respectively).

In reviewing the literature, no data was found on the association between multimorbidity and increased MPV. However, given the impact of multimorbidity on inflammation, the effect of multimorbidity on MPV can be explained in part by the proximity of thrombosis and inflammation. Although for many years platelets were recognized as a key element in thrombosis and hemostasis, more recently it has become more evident that platelet activation is also a hallmark feature in inflammation. Thus, it seems that platelets exhibit the ability to influence a wide range of seemingly unrelated pathophysiologic events [44–46]. In fact, it has been demonstrated that thrombosis and inflammation share several key molecular mechanisms and in fact are 2 intrinsically linked processes [46]. Platelet volume is determined both during megakaryopoiesis and during thrombopoiesis. Megakaryocytic maturation, platelet production, and platelet size could be regulated by cytokines, such as IL-6, granulocytes colony-stimulating factor (G-CSF), and macrophage colony-stimulating factor (M-CSF) [47]. Brown et al. [48] have demonstrated that patients with vascular disease, particularly diabetics, have an altered megakaryocyte (MK) ploidy distribution, showing a shift toward higher ploidy in association with an increased platelet mass (count × volume). Changes in platelets in diabetes probably reflected MK changes, which themselves are a response to systemic change.

Elevated MPV along with increased inflammation biomarkers (e.g., C-reactive [CRP], interleukin-6 [IL-6] among other) were reported in many conditions which are characterized by low-grade inflammation including ischemic stroke [49], utricaria [50], postoperative atrial fibrillation [51], adverse outcomes following percutaneous coronary intervention [52], myocardial infarction [53], urinary tract infection [54], exacerbations of COPD [55], hypertension [56], rheumatoid arthritis (RA) [57], osteoarthritis [58], and pneumonia [59].

In contrast, MPV was frequently found to be decreased in the presence of active high-grade inflammation; that is, active phase of RA, ankylosing spondylitis [60], ulcerative colitis, Crohn’s disease [61], and systemic lupus erythematosus (SLE) [62].

Finally, these results help to highlight the conceptual relationship between multimorbidity and platelet volume indices and corresponding worse outcomes. In many instances, the severity of MPV increase is not just linked to the type of comorbid conditions, but also should be interpreted in the context of the number of comorbid illnesses.

## Conclusions

These findings indicate that multimorbidity is associated with an increase in platelet volume indices. Multimorbidity was associated with increased platelet volume indices. MPV values increased with the increasing number of comorbid conditions with the highest platelet volume indices were observed in patients with at least four comorbid conditions. And finally, MPV values were elevated in some, but not all CVD and non-CVD conditions. More importantly, it appeared that rather disease clusters than the specific disease type were associated with increased platelet volume indices. It would seem that comorbid conditions exert synergistic rather than additive effect on MPV. This research provides a framework for the exploration of the effect of disease clusters on the outcomes rather than focusing on the specific entities.

### Study limitations

The study was carried out among patients with DM. As it frequently is a major component of multimorbidity, our results should be interpreted with caution in terms of general population. The selection of the chronic conditions for the purpose of diagnosing multimorbidity in the current study was made based on findings from previous studies that have examined the associations of these comorbidities with the prognosis of patients hospitalized with acute myocardial infarction. In addition, these data are cross-sectional, meaning that causal associations are not clear. All chronic conditions included in our study had equal weight in the principal component analysis, since we did not have any data regarding the severity or duration of a chronic disease. The small number of patients without multimorbidity could have contributed to the underdetection of meaningful differences in some of the patients’ characteristics, especially in platelet volume indices among patients with non-CVD comorbidities. We have used EDTA for anticoagulation of whole blood prior to automated cell counting. Some reports show that MPV values may increase with the use of EDTA due to platelet swelling, thus, suggesting the use of acid citrate as anticoagulant. However, most studies indicate that MPV can be measured accurately by both methods of anticoagulation—EDTA and citrate—if analysis be performed within 1 h of sampling. We did not routinely register microvascular complications (e.g., diabetic neuropathy, retinopathy, and nephropathy) which have been also reported to be associated with increased MPV [24]. In addition, we have not registered information on several patient-associated features (e.g., socioeconomic status or psychological factors) which may have influenced some of the observed associations. Despite these limitations, this is an all-comers study among a high-risk population of patients with DM and STEMI.
